# Evaluation of Meibomian gland dysfunction using meibography in patients with xeroderma pigmentosum

**DOI:** 10.5935/0004-2749.2022-0319

**Published:** 2024-02-23

**Authors:** Allexya Affonso Antunes Marcos, Denise Freitas, Rossen Mihaylov Hazarbassanov, Arthur Gustavo Fernandes, Ligia Pereira Castro, Danilo Batista Vieira de Melo, Carlos F. Martins Menck, Melina Correia Morales, José Álvaro Pereira Gomes, Rubens Belfort Neto, Arun D. Singh

**Affiliations:** 1 Department of Ophthalmology and Visual Sciences, Escola Paulista de Medicina, Hospital São Paulo, Universidade Federal de São Paulo, São Paulo, SP, Brazil; 2 Academia Nacional de Medicina, Programa Jovens Lideranças Médicas, Rio de Janeiro, RJ, Brazil; 3 DNA Repair Laboratory, Department of Microbiology, Instituto de Ciências Biomédicas, Universidade de São Paulo, São Paulo, SP, Brazil; 4 Department of Ophthalmology, Cleveland Clinic, Cleveland, US

**Keywords:** Meibomian glands/pathology, Meibomian glands/diagnostic imaging, Photography, Xeroderma pigmentosum, Eyelid diseases/diagnostic imaging, Dry eye syndromes, DNA repair, Humans, Case report

## Abstract

**Purpose:**

To assess Meibomian gland dysfunction using meibography in patients with xeroderma pigmentosum and correlate with ocular surface changes.

**Methods:**

This cross-sectional study evaluated patients with xeroderma pigmentosum. All patients underwent a comprehensive and standardized interview. The best-corrected visual acuity of each eye was determined. Detailed ophthalmic examination was conducted, including biomicroscopy examination of the ocular surface, Schirmer test type I, and meibography, and fundus examination was also performed when possible. Meibomian gland dysfunction was assessed by non-contact meibography using Oculus Keratograph^®^ 5M (OCULUS Inc., Arlington, WA, USA). Saliva samples were collected using the Oragene DNA Self-collection kit (DNA Genotek Inc., Ottawa, Canada), and DNA was extracted as recommended by the manufacturer. Factors associated with abnormal meiboscores were assessed using generalized estimating equation models.

**Results:**

A total of 42 participants were enrolled, and 27 patients underwent meibography. The meiboscore was abnormal in the upper eyelid in 8 (29.6%) patients and in the lower eyelid in 17 (62.9%). The likelihood of having abnormal meiboscores in the lower eyelid was 16.3 times greater than that in the upper eyelid. In the final multivariate model, age (p=0.001), mutation profile (p=0.006), and presence of ocular surface malignant tumor (OSMT) (p=0.014) remained significant for abnormal meiboscores. For a 1-year increase in age, the likelihood of abnormal meiboscores increased by 12%. Eyes with OSMT were 58.8 times more likely to have abnormal meiboscores than eyes without ocular surface malignant tumor.

**Conclusion:**

In the final model, age, xeroderma pigmentosum profile, previous cancer, and clinical alterations on the eyelid correlated with a meiboscore of ≥2. Meibomian gland dysfunction was common in patients with xeroderma pigmentosum, mainly in the lower eyelid. The severity of Meibomian gland dysfunction increases with age and is associated with severe eyelid changes.

## INTRODUCTION

Xeroderma pigmentosum (XP) is a genodermatosis with clinical features predominantly recognized in the dermatological, ocular, and neurological systems and is clinically characterized by cutaneous photosensitivity, pigmentary alterations, photophobia, and early development of malignancy in mucocutaneous lesions and sun-exposed ocular structures^(1)^. These manifestations are caused by cellular hypersensitivity to ultraviolet radiation (UVR), resulting from a defect in DNA repair. XP is heterogeneous, resulting from different defects in the nucleotide excision repair pathway^(2)^. It is divided into eight complementation groups, namely, XP-A, XP-B, XP-C, XP-D, XP-F, XP-G, and XP-V (XP variant), corresponding to the affected DNA repair gene. It is more common in individuals with consanguineous parents^(3)^, and changes begin in early childhood^(4)^.

Ophthalmologic abnormalities in patients with XP affect the sun-exposed tissues, periocular skin, and ocular surface^(5)^. Ocular manifestations of XP are primarily those that have been associated with UV exposure of the eyelids, cornea, or lens^(6)^. Approximately 16%-60.9% of patients with XP present eyelid alterations, with ectropion (ranging from 18% to 25%) as the most frequent, which was related to cicatricial skin alterations, mainly secondary to surgery for the treatment of eyelid and periocular skin tumors. Tear film and production are often abnormal. Dry eye syndrome (DES) occurs in 38%-100% of in XP cases^(5,7)^. Entropion, ectropion, and keratinization of the eyelid margin can lead to Meibomian gland dysfunction (MGD), which may result in the alteration of the lipid layer of the tear film. In XP, DES analysis is challenging because Schirmer’s test and ocular surface staining can be compromised by the severity of ocular surface involvement^(8)^.

In recent years, new technologies have emerged to help better evaluate ocular surface pathologies, such as the keratograph, a drop-free, contact-free, device that can measure non-invasive keratographic tear film breakup time, tear meniscus height, bulbar redness, and meibography through infrared imaging of the Meibomian glands^(9)^. For MGD evaluation, we have decided to use Arita’s meiboscore grinding to assess MGD using meibography in patients with XP and examine the correlation between ocular surface changes and XP group profile. To our knowledge, such studies have not been conducted previously.

## METHODS

### Patient recruitment

This cross-sectional study evaluated patients with XP. Some patients were already on follow-up at the Ophthalmology Department of UNIFESP’s and were invited to participate in this study. Patients from other services around Brazil were also recruited via social media (Facebook^®^ and Instagram^®^) and messaging apps (WhatsApp^®^).

All procedures involving human participants were approved by the Research Ethics Committee of the Federal University of Sao Paulo UNIFESP (#95105818.7.0000.5505) and followed the 1964 Declaration of Helsinki and its later amendments or comparable ethical standards. Patients agreed to participate in the study.

### Demographic data

All patients underwent a comprehensive and standardized interview, and obtained data were analyzed. The patients provided detailed demographic and clinical data, including sex, age, age at diagnosis, age at development of first cancer, previous medical history of skin and ocular surface malignant tumor (OSMT) confirmed by biopsy, parental consanguinity, family history, frequency of dermatological and ophthalmological follow-ups, and previous genetic testing to confirm and classify XP group profile. The patients provided details about previous treatments that could influence the ocular surface, such as topical eye treatment for ocular surface tumors and past use of systemic medications such as isotretinoin^(10)^.

### Ocular assessment

The best-corrected visual acuity (BCVA) was determined for each eye after auto-refraction, followed by subjective refraction performed by an ophthalmologist. VA measurements in the better-vision eye were categorized as follows: no visual impairment, VA ≥20/32; mild visual impairment, VA <20/32 to ≥20/63; moderate visual impairment, VA <20/63 to ≥20/200; severe visual impairment, VA <20/200 to ≥20/400; and blindness, VA <20/400. After classification, the cause of visual impairment in each eye was assessed and determined.

Detailed ophthalmic examination was conducted including biomicroscopy examination of the ocular surface, Schirmer test type I, and meibography, and fundus examination was also performed when possible. The diagnosis of active ocular surface malignant tumor (OSMT) was based on clinical examination and was supported by impression cytology and/or histopathology of biopsy specimens. AJCC Cancer Staging was used in the classification of tumors^(11)^.

The results of the Schirmer’s test, when performed, were considered normal when >10 mm and moderate--to-severe dysfunction when ≤10 mm. Non-contact meibo-graphy was performed using the Oculus Keratograph^®^ 5M (OCULUS, Inc., Arlington, WA, USA). Changes in the Meibomian glands were graded using the meiboscore^(12)^, with the following grades in each eyelid: grade 0, no loss of Meibomian glands; grade 1, loss less than one-third of the total Meibomian gland area; grade 2, loss between one-third and two-thirds; grade 3, loss of more than two-thirds (JENVIS Grading Scales for the Meibomian Glands, JenVis Research c/o Ernst Abbe University of Applied Sciences, EAH, Jena, Carl-Zeiss-Promenade 2, Jena, Germany). MGD was considered in patients with classification ≥2.

### XP-group profile

Saliva samples were collected using the Oragene DNA Self-collection kit (DNA Genotek Inc., Ottawa, Canada), and DNA was extracted as recommended by the manufacturer. A DNA targeted library was performed for next-generation sequencing using the SureSelect QXT reagent kit (Agilent Technologies, Santa Clara, CA, USA). The custom DNA repair panel was previously described^(13)^. The amplified library was sequenced using the MiSeq platform (Illumina, San Diego, CA, USA) following the manufacturer’s recommendations. Alignment and variant calling were performed using the Surecall software v3.5.1.46 (Agilent^®^) and GRCh37/hg19 human genome reference (University of California Santa Cruz). The mutations were classified according to the American College of Medical Genetics (ACMG) guideline^(14)^.

### Statistical methodology

Statistical analysis of the correlation of ocular surface changes with meibography findings was performed only in patients who underwent this examination. Initially, data were analyzed descriptively. For categorical variables, absolute and relative frequencies were presented, and for numerical variables, summary measures were used.

To evaluate the meiboscore classification, as they present measurements by side and eyelid position, generalized estimating equation (GEE) models with logit link function and binomial marginal distribution were used. The GEE approach, which consists of a generalization of generalized linear models, allows the incorporation of the dependence between observations in the same patient. In the regression models, univariate and multivariate models were adjusted. Significant predictor variables at 20% in the univariate analysis were selected in the initial multivariate model. Then, non-significant variables at 5% were excluded individually in order of significance (backward method). For all statistical tests, a significance level of 5% was adopted. GHG models were estimated using STATA 12. For other analyses, IBM SPSS Statistics version 20.0 (IBM Corp., Armonk, NY, USA) was used.

## RESULTS

### Patient recruitment

Seven (17%) patients were already being followed up at the ophthalmology outpatient clinic of Hospital São Paulo before the start of this study, and the other 35 (83%) patients were recruited through social media.

### Demographic data

The study enrolled a total of 42 participants; 29 (69%) patients were female. The mean age at the examination was 26.5 (range, 2.00-62.00; median, 24) years. The mean age at XP diagnosis was 8.5 (range, 0.33-30.00; median, 5) years (Table 1).

**Table 1:** Demographic data and XP-group profile

Age at exam (years) N=42 patients		
<18	14	33%
19-40	17	41%
>41	11	26%
XP^*^-group profile N =42 patients		
XPC	20	47%
XPV	7	17%
XPE	6	14%
XPD	2	5%
Unknown	7	17%
Duration since diagnosis (years) N =42 patients		
<10	11	26%
11-30	23	55%
>30	8	19%
Best corrected visual acuity (BCVA) N = 40 patients		
No visual impairment (VA ^†^ ≥20/32)	15	37%
Mild visual impairment (VA ^†^ <20/32 to ≥20/63)	13	32%
Moderate visual impairment (VA ^†^ <20/63 to ≥20/200)	6	15%
Severe visual impairment (VA ^†^ <20/200 to ≥20/400)	1	2%
Blindness (VA ^†^ <20/400)	5	12%

* XP= xeroderma pigmentosum.

† VA= visual acuity.

The mean age at the first cancer diagnosis was 10.24 (range, 1.00-30.00, median, 8) years. Moreover, 28 (67%) patients had consanguineous parents, 26 (62%) had a positive family history of XP, 39 (93%) had skin cancer, and 50% had not received local treatment for eye cancer (such as surgical excision, cryotherapy, brachytherapy, and topical medical therapy - topical chemotherapy and immunotherapy). No patient had previously used isotretinoin. Dermatologists regularly followed 23 (55%) patients, and ophthalmologists followed 29 (69%) patients (Table 2).

**Table 2:** Clinical characteristics and correlation with abnormal meiboscore.

	Meiboscore	
	<2	≥2
**Sex**		
Female	29 (54,7)	35 (87,5)
Male	24 (45,3)	5 (12,5)
**Age (years), mean ± SD**	26,2 ± 17,7	35,7 ± 17,9
**Consanguinity**		
No	17 (32,1)	19 (47,5)
Yes	36 (67,9)	21 (52,5)
**Family history**		
No	27 (50,9)	8 (20,0)
Yes	26 (49,1)	32 (80,0)
**Mutation profiling**		
XPC	14 (32,6)	17 (56,7)
XPD	6 (14)	2 (6,7)
XPE	4 (9,3)	6 (20)
XPV	19 (44,2)	5 (16,7)
**History of cutaneous tumor**		
No	1 (1,9)	7 (17,5)
Yes	52 (98,1)	33 (82,5)
**Care status: Regular Follow up Dermatology**		
No	15 (28,3)	23 (57,5)
Yes	38 (71,7)	17 (42,5)
**Care status: Regular Follow up Ophthalmology**		
No	20 (37,7)	5 (12,5)
Yes	33 (62,3)	35 (87,5)
**BCVA**		
No visual impairment VA ≥20/32	39 (73,6)	25 (62,5)
Mild visual impairment VA <20/32 to ≥20/63	8 (15,1)	2 (5,0)
Moderate visual impairment VA <20/63 to ≥20/200	6 (11,3)	9 (22,5)
Blindness VA <20/400	0 (0,0)	4 (10,0)
**Active Eye Cancer**		
No	42 (80,8)	34 (91,9)
Yes	10 (19,2)	3 (8,1)
**Previous Eye Cancer**		
No	47 (90,4)	21 (56,8)
Yes	5 (9,6)	16 (43,2)
**Eyelid**		
Normal	9 (17,0)	7 (17,5)
Anormal	44 (83,0)	33 (82,5)
**Melanocytic lesions of skin**		
No	22 (41,5)	24 (60,0)
Yes	31 (58,5)	16 (40,0)
**Madarosis**		
No	52 (98,1)	29 (72,5)
Yes	1 (1,9)	11 (27,5)
**Conjunctiva**		
Normal	2 (3,8)	2 (5,0)
Anormal	51 (96,2)	38 (95,0)
**Hyperemia**		
No	19 (35,8)	20 (50,0)
Yes	34 (64,2)	20 (50,0)
**Pterygium**		
No	43 (81,1)	31 (77,5)
Yes	10 (18,9)	9 (22,5)
**Cornea**		
Normal	24 (45,3)	12 (30,0)
Anormal	29 (54,7)	28 (70,0)
**Corneal scar or neovascularization**		
No	40 (75,5)	20 (50,0)
Yes	13 (24,5)	20 (50,0)
**Schirmer - classification**		
Anormal (≤10 mm)	4 (16,0)	7 (33,3)
Normal (>10mm)	21 (84,0)	14 (66,7)

The table contains information on the 27 patients who underwent meibography. In addition, the analysis correlates the eyelid (upper or lower, right or left) with the demographic data.

### XP group profile

Mutation testing revealed XPC as the most frequent genotype (n=20, 47%), and XPD being the rarest variant (n=2, 5%) (Table 1). XP group profile was unknown in 7 (17%) patients (the sample collected was insufficient for analysis).

### Ocular assessment

**Figure 1:**
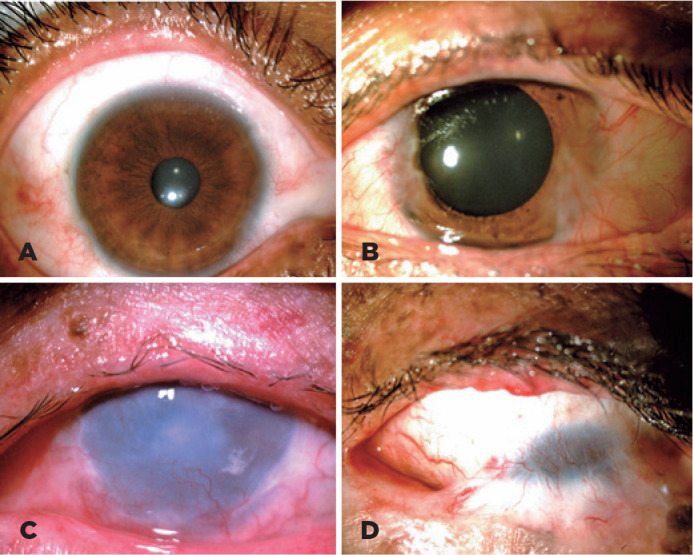
Ocular findings in patients with xeroderma pigmentosum. A 34-year-old woman with XPE and normal anterior-segment examination (A). A 32-year-old man with XPE and nasal and temporal pterygium (B). A 23-year-old woman with XPC, madarosis of upper eyelid lashes, absence of lower eyelid, conjunctival hyperemia with nasal pterygium, and corneal opacity with neovascularization (C). A 26-year-old woman with XPC and symblepharon affecting the inferior tarsal conjunctiva to the superior limbus (D).

The BCVA could be recorded reliably only in 40 participants. Two pediatric patients had no VA data because they refused VA measurements. In this population, the main cause of visual impairment and blindness were refractive error (32%), corneal scar (30%), and amblyopia (12%) (Table 1). Pathologic changes affected the eyelid, conjunctiva, and cornea in 81%, 74%, and 67% of participants, respectively (Figure 1).

**Figure 2:**
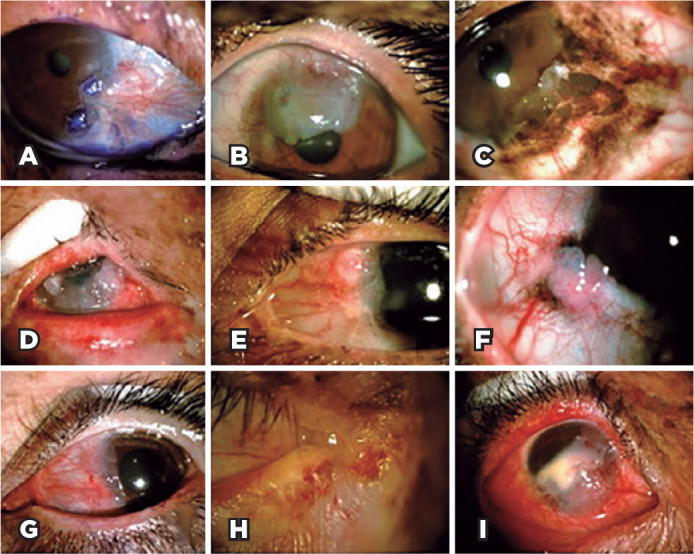
Ocular surface tumors in patients with xeroderma pigmentosum. A 61-year-old woman with XPE and ocular surface squamous neoplasia (OSSN) staining with toluidine blue (A). A 21-year-old man with XPV and OSSN affecting the cornea (B). An 18-year-old woman with XPD and conjunctiva melanoma affecting the conjunctiva and cornea (C). A 23-year-old woman with XPC and OSSN affecting the conjunctiva and cornea (D). A 23-year-old man with XPV and OSSN affecting the conjunctiva (E). An 18-year-old woman with XPD and OSSN affecting the conjunctiva (F). A 20-year-old woman with XPV and OSSN affecting the conjunctiva (G). An 18-year-old woman with XPC and basal cell carcinoma of the eyelid (H). A 9-year-old girl with XPC and OSSN affecting the conjunctiva and cornea (I).

Twelve participants (29%) presented with an active ocular tumor during the clinical examination (Figure 2). Active OSMTs were basal cell carcinoma of the eyelid (1) (TIN0M0), conjunctiva melanoma (1) (T1b), and 10 cases of ocular surface squamous neoplasia (OSSN). One patient had simultaneous squamous cell carcinoma in the right eye and conjunctival melanoma in the left eye. Fifteen patients underwent Schirmer’s test (35%), 12 of them in both eyes, and 3 in only one eye, and abnormal results were obtained in 6 (33%) eyes.

Posterior fundus evaluation was not possible in seven patients because of corneal opacity. The evaluation included two patients with a choroidal nevus and one with hypertensive retinopathy.

### Meiboscore

**Figure 3:**
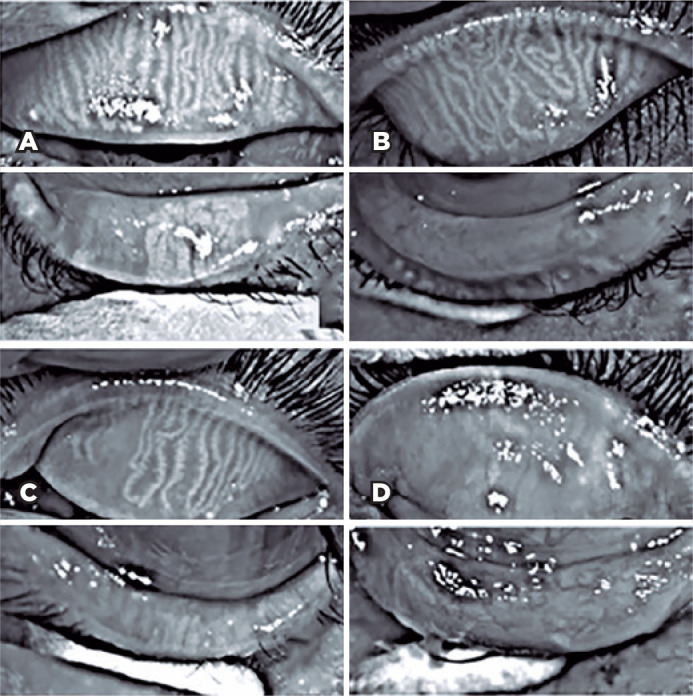
Meibography observations in patients with xeroderma pigmentosum. Upper and lower eyelids of the right eye of a 10-year-old girl with XPC. In the upper eyelid, the meiboscore was 0, which indicated no loss of Meibomian glands; in the lower eyelid, the meiboscore was 1 (A, loss less than one-third). A 10-year-old girl with XPC. In the upper eyelid of the right eye, the meiboscore was 0. The meiboscore was 3 in the lower eyelid (B, area of loss more than two-thirds). A 50-year-old woman with XPV and the meiboscores were 2 (area loss was between one-third and two-thirds) in the upper eyelid and 3 in the lower eyelid (C). A 20-year-old woman with XPC having a meiboscore of 3 in both eyelids (D).

In this study, 27 patients underwent meibography, 24 of them in both eyes, and 3 in only one eye, totaling 51 eyes. The meiboscore was abnormal (≥2) in the upper eyelid of 8 (29.6%) patients and in the lower eyelid of 17 patients (62.9%) (Figure 3).

### Correlation results

Statistical analysis to correlate ocular surface changes with meibography findings was performed only in 27 patients who underwent this examination. Variables such as sex, age, XP group profile, OSMT, and abnormal eyelid (significant at 20% in the univariate models) were used for the initial multivariate model. In the final model, age (p=0.001), XP group profile (p=0.006), OSMT (p=0.014), and lower eyelid (p<0.001) remained significant for abnormal meiboscores (Table 3).

**Table 3:** Statistical analysis of factors associated with abnormal Meiboscore

	Univariate Model		Multivariate Model	
	Gross odds ratio (95% CI)	p	Adjusted odds ratio (95% CI)	p
Male (ref.^*^ = Female)	0.19 (0.04 - 0.94)	0.04	-	-
Age (years)	1.02 (0.99 - 1.06)	0.18	1.12 (1.04 - 1.20)	0.001
XP-group profile (ref.^*^ = XPC)		0.18		0.006
XPD	0.20 (0.01 - 3.39)	0.26	0.007 (0.000 - 0.204)	0.004
XPE	1.37 (0.16 - 11.72)	0.77	0.035 (0.003 - 0.380)	0.006
XPV	0.16 (0.02 - 1.11)	0.06	0.003 (0.000 - 0.095)	0.001
BCVA^†^ VA^‡^ <20/32 (ref.^*^ VA^‡^ ≥20/32)	2.34 (0.59 - 9.38)	0.23	-	-
Eye surface malignant tumor §	2.30 (0.75 - 7.05)	0.15	58.76 (2.28 - 1.514.70)	0.014
Abnormal eyelids (ref.^*^ = normal)	2.20 (0.72 - 6.72)	0.17	-	-
Conjunctiva - abnormal (ref.^*^ = normal)	0.47 (0.08 - 2.73)	0.40	-	-
Cornea - abnormal (ref.^*^ = normal)	1.64 (0.62 - 4.35)	0.32	-	-
Schirmer - abnormal (ref.^*^ = normal)	1.01 (0.68 - 1.50)	0.96	-	-
Lower eyelid Meiboscore (ref.^*^ = upper)	3.16 (1.88 - 5.31)	<0.001	16.29 (3.34 - 79.36)	<0.001

* Ref= reference.

† BCVA= best-corrected visual acuity.

‡ VA= visual acuity.

§ History or presence of eye surface malignant tumor.

Thus, advancing age increased the chance of having a meiboscore ≥2 by (12% for each 1-year increase). Furthermore, patients with XPD, XPE, and XPV had lower odds than patients with XPC (99.3%, 96.5%, and 99.7% lower, respectively). In addition, eyes with OSMT were 58.8 times more likely to have abnormal meiboscores than eyes without OSMT. The risk of having abnormal meiboscores in the lower eyelid was 16.3 times greater than that in the upper eyelid.

## DISCUSSION

In Brazil, approximately 200 patients have XP, which would represent one case per million inhabitants^(15)^. Therefore, our sample represents approximately 25% of the nationwide Brazilian patient cohort. Social networks were an essential tool in recruiting these patients spread across the country, and other reports have already demonstrated the critical role of this tool in the public health strategy^(16)^.

This study has an essential representativeness in the universe of a rare disease. However, it simultaneously presents limitations in its statistical correlations because of the small sample. In addition, the study is limited by severe alterations on the ocular surface of patients with XP, and it was impossible to perform a reliable Schirmer I test. Of the 27 patients included in this study, only 15 could be considered for our analysis. Similarly, only 27 of the 42 patients with XP underwent meibography because many patients had undergone surgeries that required complete excision of the eyelid and fibrosis and scars did not allow the eyelid to be everted for the examination. Only the 27 patients who underwent meibography examination were considered for the statistical analysis.

Systemic treatments include the administration of 13-cis-retinoid acid (isotretinoin), and high-dose oral isotretinoin is effective as chemoprophylaxis of non--melanoma skin cancers in patients with XP^(17)^ but can cause atrophy of the Meibomian glands^(18)^. None of the patients in our cohort disclosed isotretinoin used.

Ocular diseases in XP are almost exclusively limited to the anterior, UV-exposed structures of the eye, namely, the eyelids, conjunctiva, and cornea^(7)^. Except for two case reports that have demonstrated subclinical retinal changes in a patient with XP-A and XP-D on postmortem histopathology, retinal abnormalities have not been observed in XP^(19)^. Similarly, optic atrophy is not a known hallmark of XP and has only ever been described in one case, where it may have been a coincidental finding^(19)^. Our findings corroborate these premises because no retinal changes were observed in our patients.

Previous studies on ocular surface changes have reported signs of dry eye disease in 38%-100% of patients based on the methods used for assessment such as the tear film breakup time or Schirmer’s test^(5,20,21)^. MGD signs were recorded as an abnormal meiboscore (<2) in 60% and 56% of the upper and lower eyelids, respectively^(22)^. To our knowledge, such studies have not been reported previously. The severity of MGD (abnormal meiboscores) correlated significantly with age (p=0.001), worsening by 12% for each 1-year increase in age. Such a relationship probably reflects cumulative exposure to UVR, an important known risk factor of clinical manifestations and disease severity in patients with XP^(23)^.

In general, the lower eyelid is more frequently abnormal than the upper eyelid as evidenced by the loss of lashes documented previously^(21)^. A worse meiboscore in the lower eyelid than in the upper eyelid can be explained by the UVR protection by the eyebrow afforded to the upper lid^(24)^. Moreover, frequent involvement of the lower eyelid may result from the light reflection by the cornea onto the lower lid margin^(24)^. Alternatively, the presence of tight skin causing ectropion in the lower eyelid can explain the predominance of lower eyelid involvement^(7)^.

In addition, abnormal meiboscores were also associated with the XP group profile (p=0.006), patients with XPD, XPE, and XPV mutation had lower odds than patients with XPC mutation (99.3%, 96.5%, and 99.7% lower, respectively). Although several factors may influence the development and function of the Meibomian glands, previous reports on human and mouse models have demonstrated that diminished meibocyte differentiation, renewal, and gland size and increased inflammatory cell infiltration^(25)^ are related to decrea-sed expression of peroxisome proliferator-activated receptor-gamma^(26)^, a factor that mediates adipose tissue hypoplasia in the XP-D subtype of XP^(27)^. Moreover, XPA-deficient mice exposed to low daily doses of UV-B radiation can develop irritated eyelid margins^(28)^. Studies on a larger number of patients are needed to confirm this genotype-phenotype association.

The presence of MGD can be a significant contributor to ocular surface inflammation and resulting deficits in visual function^(29)^. The importance of abnormal meiboscore relies on its association with overall severe ocular surface changes and most importantly with the presence or past OSMT history. Eyes with OSMT were nearly 60 times more likely to have abnormal meiboscore than eyes without OSMT. Although MGD may not have directly contributed to the pathogenesis of OSMT, the presence of MGD is an objectively assessed biomarker for severe ocular surface diseases and a risk of OSMT development. An abnormal Meiboscore was noted in patients as young as 9 years and even in the upper eyelids, suggesting that MGD is a primary abnormality rather than secondary to ocular surface changes. As a corollary, our observations indicate a new line of intervention, that is, incorporation of MGD management in the overall care plan of patients with XP^(29)^.

In the final model, age, XP group profile, previous cancer, and clinical alteration on the eyelid correlated with a meiboscore of ≥2. DGM was frequent in patients with XP, mainly in the lower eyelid. The severity of MGD increases with age and is associated with severe eyelid changes.
